# Prognostic Role of the Total Psoas Area Index (TPAI) and Its Association with Survival After Gastric Cancer Surgery

**DOI:** 10.3390/cancers18071135

**Published:** 2026-04-01

**Authors:** Manuel José Torres-Jurado, Omar Abdel-Lah-Fernández, María Alejandra Arévalo-González, Laura Vicente-González, Nerea García-González, Francisco José Mena Valle, Juan Manuel Nieto-Arranz, María Lourdes Hernández-Cosido, Felipe Carlos Parreño-Manchado, Francisco Blanco-Antona

**Affiliations:** 1 General and Digestive Surgery Department, University Hospital of Salamanca, 37007 Salamanca, Spainngarciagonzalez1@saludcastillayleon.es (N.G.-G.); fjmena@saludcastillayleon.es (F.J.M.V.); jmnieto@saludcastillayleon.es (J.M.N.-A.); fparreno@usal.es (F.C.P.-M.);; 2 Radiodiagnostic Department, University Hospital of Salamanca, 37007 Salamanca, Spain; 3 Department of Statistics, Faculty of Medicine, University of Salamanca, 37007 Salamanca, Spain; laura20vg@usal.es

**Keywords:** gastric cancer, Sarcopenia, total psoas area index, computed tomography, survival

## Abstract

Objectives: Gastric cancer is one of the leading causes of cancer-related mortality. Sarcopenia, characterized by reduced skeletal muscle mass, strength, and physical performance, is associated with poorer outcomes in gastrointestinal cancers. This study aimed to evaluate the prevalence of sarcopenia, including the Total Psoas Area Index (TPAI), and its association with tumor stage, postoperative complications, and survival in patients with gastric adenocarcinoma. Methods: A retrospective study was conducted, including 79 patients who underwent surgery for gastric adenocarcinoma. Sarcopenia was assessed using a combination of CT-derived muscle mass (TPAI), muscle strength (dynamometry), and functional status (SARC-F). Patients were divided into sarcopenic (SG) and non-sarcopenic (NSG) groups. Clinical and survival data were compared between groups. Results: Sarcopenia was identified in 36.7% of patients. Early-stage disease (I–II) was less frequent in the SG (20.7%) compared to the NSG (44%), whereas stage IV tumors were more common in the SG (20.7% vs. 6%). Sarcopenic patients had a lower body mass index and underwent fewer laparoscopic procedures (44.8% vs. 68%, *p* = 0.03). Postoperative complications, such as pneumonia and ileus, were more frequent in the SG. Overall survival was significantly lower in the SG (17 vs. 21 months, respectively, *p* = 0.042), with a reduction in disease-free survival as well. Postoperative TPAI values remained lower in the SG. Conclusions: Sarcopenia, as defined by combined assessment including TPAI, was associated with advanced tumor stage, increased postoperative risk, and reduced survival in patients with gastric cancer. Preoperative assessment of muscle mass may help identify high-risk patients and optimize clinical management.

## Simple Summary

Gastric cancer is one of the leading causes of cancer-related death worldwide. In recent years, loss of muscle mass, known as sarcopenia, has been recognized as an important factor affecting patient outcomes. In this study, we evaluated muscle mass, strength, and functional status. We found that patients with sarcopenia were more likely to have advanced disease, experience more postoperative complications, and have lower survival rates. These results suggest that assessing muscle mass before surgery could help identify high-risk patients and guide personalized treatment strategies, such as nutritional support or prehabilitation, to improve outcomes.

## 1. Introduction

Gastric cancer is the fifth-most common malignancy and the third leading cause of cancer-related death worldwide [1]. Despite advances in diagnosis and treatment, it remains a major global health challenge due to its biological heterogeneity, frequent late-stage presentation, and variable response to therapy. In addition to tumor-related factors, growing evidence suggests that the tumor microenvironment, including neural signaling, stromal interactions, and immune modulation, plays a crucial role in tumor progression and metastasis [2,3,4]. Furthermore, sarcopenia has been consistently associated with adverse perioperative and oncological outcomes in patients undergoing gastrectomy [5]. Traditionally, sarcopenia has been defined as a syndrome characterized by progressive and generalized loss of skeletal muscle mass, strength, and physical performance [6]. According to the European Working Group on Sarcopenia in Older People (EWGSOP), the diagnosis of sarcopenia requires not only reduced muscle quantity or quality, but also decreased muscle strength and/or impaired physical performance [6,7].

However, in routine clinical oncology practice, muscle mass is frequently assessed using imaging techniques such as computed tomography (CT), which are routinely performed for staging and follow-up. CT-based body composition analysis, particularly at the level of the third lumbar vertebra (L3), has been widely adopted as a reliable and reproducible method for estimating skeletal muscle mass [8,9].

Among the different radiological parameters, the total psoas area index (TPAI) has gained attention as a simple and accessible surrogate marker of muscle mass. Several studies have consistently demonstrated that low CT-derived muscle mass is associated with increased postoperative complications, reduced tolerance to oncological treatments, and poorer survival in patients with gastrointestinal malignancies, including gastric cancer [10,11,12].

Despite the growing interest in this field, important limitations remain. First, there is considerable heterogeneity in the cut-off values used to define low muscle mass, which vary across populations and cancer types. Second, most studies rely on cross-sectional preoperative assessments, with limited data on the longitudinal evolution of muscle mass after surgery. Third, CT-derived muscle measurements are frequently used as a proxy for sarcopenia in oncological research; however, they do not fully capture the multidimensional nature of this syndrome, as they do not include functional parameters. Despite these advances, the prognostic role of integrated sarcopenia assessment combining CT-derived muscle mass with functional parameters remains insufficiently explored in gastric cancer.

In addition to surgical and oncological treatment, multidisciplinary management—including early nutritional assessment and intervention—plays a crucial role in the care of patients with gastric cancer. The involvement of a clinical nutrition specialist within the multidisciplinary team has been increasingly recognized as a key component in optimizing perioperative outcomes and long-term prognosis in patients with gastrointestinal malignancies. Nutritional status is closely linked to muscle mass and functional reserve, and its optimization may contribute to reducing postoperative complications and improving overall outcomes. Recent evidence supports the integration of nutritional strategies within perioperative care pathways in gastrointestinal oncology [13].

In this context, the present study aims to evaluate CT-defined muscle mass using the total psoas area index (TPAI) and to investigate its association with tumor stage, postoperative complications, and survival in patients undergoing curative-intent surgery for gastric cancer. Furthermore, we explore the longitudinal evolution of TPAI during the first postoperative year. By focusing on an objective and routinely available imaging parameter, this study seeks to provide clinically applicable information that may improve preoperative risk stratification and support personalized management strategies in gastric cancer patients.

## 2. Materials and Methods

### 2.1. Study Population

A retrospective observational study was conducted, including patients diagnosed and treated for gastric cancer with curative intent between February 2021 and December 2023 at the Department of General and Digestive Surgery, University Hospital of Salamanca (HUSA). A total of 79 patients were included, all of whom had complete records of preoperative abdominal and pelvic computed tomography (CT) imaging, as well as follow-up CT scans at 6 and 12 months postoperatively. All procedures were performed by the same surgical team. Exclusion criteria included patients under 18 years of age, non-oncologic gastric pathology, patients with known metastatic disease at initial preoperative staging, prior oncologic gastric surgery, or local recurrence. However, some patients were upstaged to stage IV based on postoperative pathological findings.

The following variables were analyzed: demographic data; comorbidity assessed using the age-adjusted Charlson Comorbidity Index (CCI) [14] and the American Society of Anesthesiologists (ASA) classification; TNM staging system (eighth edition) [15]; preoperative laboratory data; histological type; sarcopenia screening using the SARC-F questionnaire [16]; anthropometric markers; appendicular skeletal muscle mass index (ASMI) [17]; surgical approach and procedure; postoperative complications graded according to the Clavien–Dindo classification [18] and the Comprehensive Complication Index (CCI) [19]; hospital stay; 3-month readmission or reoperation; systemic treatment; overall survival (OS); and disease-free survival (DFS).

Perioperative management followed institutional protocols based on enhanced recovery after surgery (ERAS) principles, including early mobilization and nutritional assessment. Nutritional status was evaluated as part of routine clinical care; however, these variables were not included in the present analysis, as the primary objective of this study focused on CT-based muscle mass assessment (TPAI).

Histopathological diagnosis was based on the 2019 revision of the World Health Organization (WHO) classification [20]. The study protocol was approved by the local ethics committee (CEIm: PI 2025 02 1832), including approval for the use and publication of retrospectively analyzed data.

### 2.2. Definition of Sarcopenia by CT

The total psoas area (TPA) at the level of the third lumbar vertebra (L3), which correlates with whole-body skeletal muscle mass, was selected for analysis. An axial CT image at the L3 level was used to measure the cross-sectional area of the psoas muscle (TPA = left + right psoas muscle area) [9]. The total psoas area index (TPAI) was calculated using the formula: TPAI = total psoas muscle area (cm^2^)/height^2^ (m^2^) [21]. Psoas muscle density (PMD) was defined as the mean attenuation of the psoas muscle in Hounsfield units (HU) [21,22].

**Figure 1:**
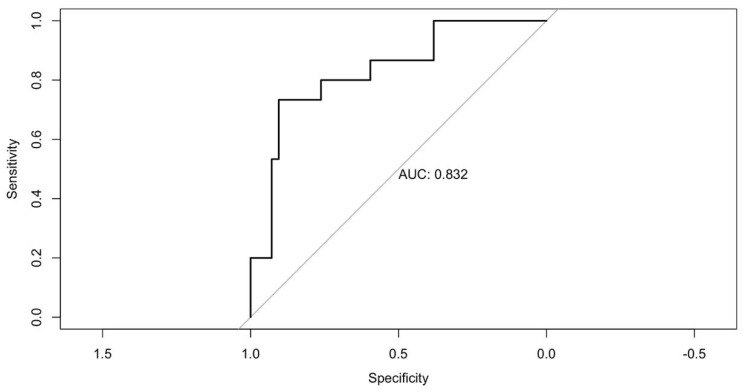
Receiver operating characteristic (ROC) curve of the total psoas area index (TPAI) for predicting mortality. The area under the curve (AUC) was 0.832, indicating good discriminative ability. The optimal cut-off values were determined using the Youden index. The diagonal line represents the line of no discrimination.

CT scans performed preoperatively, at 6 months, and at 12 months postoperatively were analyzed. A receiver operating characteristic (ROC) curve was generated using mortality as the outcome variable to determine the optimal TPAI cut-off point for each sex (Figure 1). Sarcopenia was defined according to the EWGSOP2 criteria, integrating muscle mass, muscle strength, and functional performance. Muscle mass was assessed using the CT-derived total psoas area index (TPAI), muscle strength by handgrip dynamometry, and functional status using the SARC-F questionnaire. Patients were classified as sarcopenic when low muscle mass (based on TPAI) was accompanied by reduced muscle strength and/or impaired functional status.

### 2.3. Statistical Analysis

Quantitative variables were summarized as means and standard deviations or medians and interquartile ranges (IQR), as appropriate, while categorical variables were expressed as frequencies and percentages. The ROC-derived TPAI cut-off was used to define low muscle mass, which was subsequently integrated with muscle strength and functional parameters to establish sarcopenia according to EWGSOP2 criteria [23]. The optimal cut-off point was identified using the Youden index to maximize both sensitivity and specificity.

Group comparisons were performed using the Wilcoxon rank-sum test for continuous variables and Pearson’s chi-squared test or Fisher’s exact test for categorical variables, as appropriate.

Overall and disease-free survival were evaluated using Kaplan–Meier survival curves, and differences between groups were assessed using the log-rank test. Multivariate analysis of factors associated with survival was conducted using a Cox proportional hazards regression model, adjusting for clinically relevant covariates and those with significant associations in univariate analysis.

All statistical analyses were performed using R Core Team. R: A Language and Environment for Statistical Computing; R Foundation for Statistical Computing: Vienna, Austria, 2024. Version 4.5.2. [24], utilizing the packages survival [25], survminer [26], and pROC [27]. A *p*-value of < 0.05 was considered statistically significant.

## 3. Results

Sarcopenia classification was based on combined assessment of muscle mass, strength, and functional status according to EWGSOP2 criteria.

Table 1 summarizes the clinicopathological characteristics of the entire sample. A total of 79 patients were analyzed during the study period, of whom 63% were male. The median age was 74 years (interquartile range: 65–83 years). Comorbidity burden, assessed using the age-adjusted Charlson Comorbidity Index (ACCI), revealed that the majority of patients were at elevated risk.

Specifically, 1.2% of patients were classified as low risk (0–1 points), 7.6% as moderate risk (2–3 points), 31.6% as high risk (4–5 points), and 59.5% as very high risk (≥6 points). According to the ASA classification, 50.6% of patients had severe systemic disease. All patients were diagnosed with gastric adenocarcinoma.

The most frequent tumor location was the antrum (50.6%), followed by the body (22.8%), cardia (17.7%), and fundus (8.9%). Neoadjuvant chemotherapy was administered to 38% of patients. According to the SARC-F screening tool, 24% of patients were at high risk for sarcopenia.

The ASMI showed that 44% of patients had low muscle mass based on sex-specific thresholds. The preoperative CT-based total psoas area index (TPAI) was 6.33 (range: 4.8–7.8), with 64.6% of patients classified in the non-sarcopenic group (NSG) and 36.7% in the sarcopenic group (SG).

**Figure 2:**
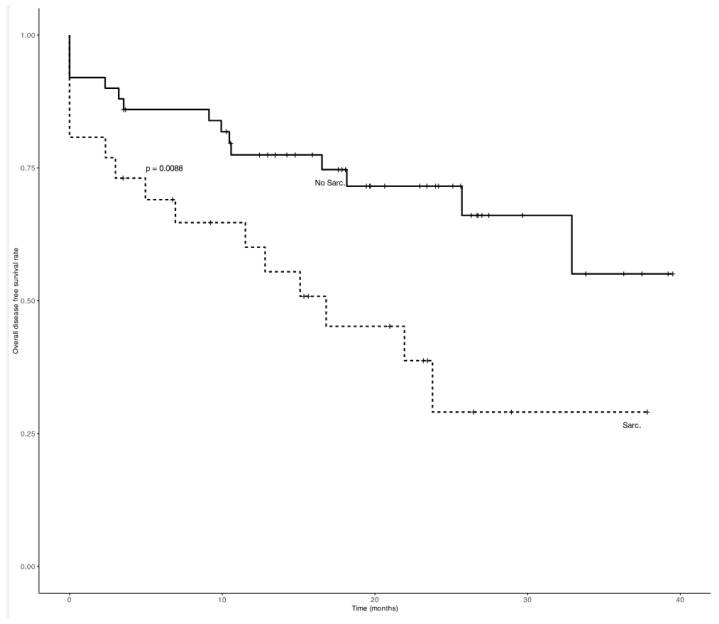
Kaplan-Meier curves for overall survival. Analysis of subgroups based on the presence of sarcopenia, defined according to EWGSOP2 criteria. “+” indicates censored observations.

The optimal TPAI cut-off point determined by ROC curve analysis was 6.04 for men and 4.98 for women (Figure 2). Table 1 summarizes the characteristics of the surgical intervention and postoperative outcomes. A total of 15% of interventions (12 patients) were performed urgently.

Regarding the type of surgery, 65.8% underwent partial gastrectomy—of which 54.4% were Roux-en-Y and 11% Billroth II—while 34% underwent total gastrectomy. The surgical approach was laparoscopic in 59% of cases, open in 32%, and required conversion to open surgery in 8.9%.

Regarding complications, according to the Clavien–Dindo classification, 42% of patients experienced no complications, and 34% experienced mild complications (grades I–II). Severe complications were observed in 19% of patients, with 5.1% having grade IIIA complications and 2.5% grade IIIB. The most severe complications (grade IV) affected 11.4% of patients, with 6.3% grade IVA and 5.1% grade IVB.

The Comprehensive Complication Index (CCI) was 22 (range: 29), and 56% of patients experienced some form of postoperative complication. The most frequent complications were paralytic ileus (27%), pleural effusion (15%), intra-abdominal collections (19%), pneumonia (11%), gastrojejunal anastomotic leak (11%), and duodenal stump leak/fistula (10%).

The average postoperative hospital stay was 13 days. The readmission rate within 90 days was 21%, and 17% of patients required reoperation in the postoperative period.

According to the TNM staging system, 20.2% of patients were stage IA and 17.7% stage IB. More advanced stages, such as IVB, affected 7.6% of patients. In terms of resection status, 14% had microscopic residual tumor (R1) and 3.8% had macroscopic residual tumor (R2). Some patients were upstaged to stage IV based on postoperative pathological findings.

Adjuvant chemotherapy was administered to 56% of patients according to standard clinical indications. The mean overall survival (OS) was 43.7 months (95% CI: 42.6, 44.8), while disease-free survival (DFS) was 24.9 months (range: 22.7, 27.1). The median follow-up was 18 months.

During this period, 30% of patients died. The main cause of death was disease recurrence (26.6%), followed by complications (6.3%) and other causes (5.1%).

Table 2 shows the comparisons between NSG and SG patients based on TPAI. No significant differences were found in age between the two groups (*p* = 0.2). Sex distribution was also similar, with 62% males in the NSG and 65.5% in the SG (*p* = 0.8).

A significant difference in body mass index (BMI) was observed between the groups (*p* < 0.001). The NSG had a higher prevalence of obesity (24%) and a lower prevalence of underweight (4%), whereas the SG showed a higher proportion of underweight patients (17.2%) and no cases of obesity.

ASMI analysis showed that 65.5% of patients in the SG had low ASMI, compared to 32% in the NSG (*p* = 0.002).

Regarding comorbidities, all patients in both groups had high comorbidity scores (≥3 on the CCI), with no significant differences between groups (*p* = 0.2). No differences were observed in ASA classification (*p* = 0.6) or histological cancer type (*p* = 0.3).

A notable difference was observed in tumor stage at diagnosis between sarcopenic and non-sarcopenic patients. Among the NSG, 44% were diagnosed at early stages (I–II), 50% at locally advanced stage (III), and only 6% at stage IV. In contrast, the SG had a markedly higher proportion of patients with advanced disease: only 20.7% were at stages I–II, 58.6% at stage III, and 20.7% at stage IV.

Patients in the NSG underwent laparoscopic surgery more frequently (68%) than those in the SG (44.8%) (*p* = 0.03). Additionally, 92% of NSG patients had scheduled surgeries, compared to 72.4% in the SG.

No significant differences were observed in the overall postoperative complication rate (*p* = 0.12). Sarcopenic patients showed higher rates of pneumonia (17.2% vs. 8%, *p* = 0.22) and paralytic ileus (34.5% vs. 22%, *p* = 0.31), although these differences did not reach statistical significance.

**Figure 3:**
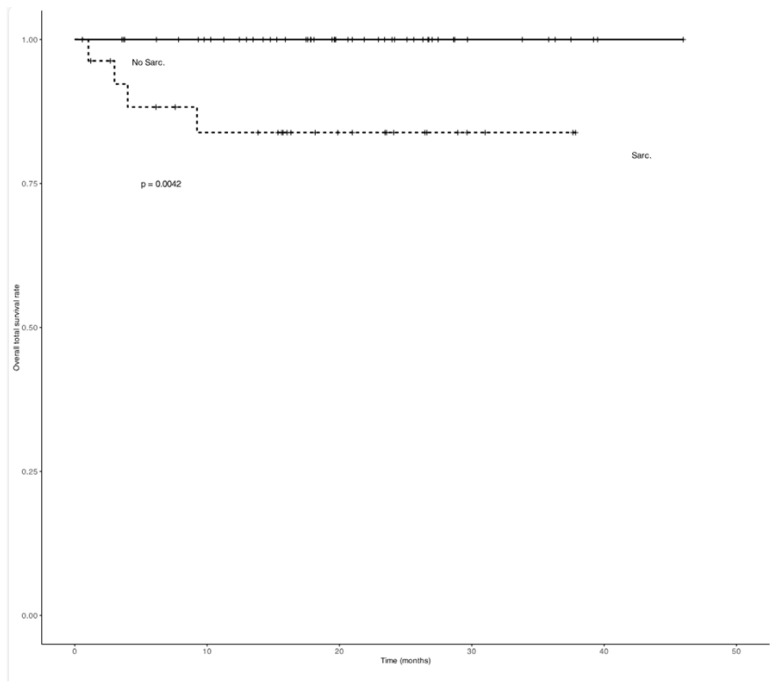
Kaplan-Meier curves for disease free survival rate. Analysis of subgroups based on the presence of sarcopenia, defined according to EWGSOP2 criteria. “+” indicates censored observations.

Regarding survival, NSG patients had significantly higher OS (21 months, range 11–32) compared to SG patients (17 months, range 6–28) (*p* = 0.042). DFS was also lower in SG patients, approaching statistical significance (*p* = 0.055). The SG showed a significantly steeper decline in survival probability compared to the NSG (*p* = 0.0042) (Figure 2 and Figure 3).

A Cox proportional hazards regression analysis was performed to evaluate predictors of overall survival. In the multivariate model, Sarcopenia (EWGSOP2-defined) was independently associated with worse survival (HR: 1.8; 95% CI: 1.1–3.2; *p* = 0.03), along with advanced tumor stage (HR: 2.5; 95% CI: 1.4–4.5; *p* = 0.002). Age and BMI were not independently associated with overall survival in the multivariate model. Figure 3 shows a significantly greater decrease in the cumulative probability of remaining disease-free in the SG compared to the NSG (*p* = 0.0055).

A Cox proportional hazards regression analysis was performed to evaluate predictors of overall survival (Table 3). A Cox proportional hazards analysis was also performed for disease-free survival (DFS). In the univariate analysis, sarcopenia was significantly associated with reduced DFS (HR: 2.54; 95% CI: 1.24–5.23; *p* = 0.01). However, this association did not remain statistically significant in the multivariate model adjusted for age, sex, and tumor stage (HR: 1.57; 95% CI: 0.70–3.51; *p* = 0.27) (Table 4).

**Figure 4:**
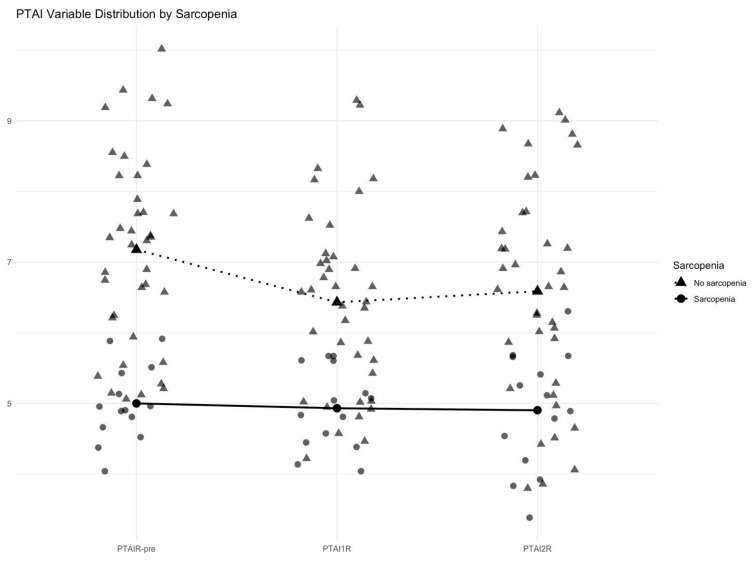
Illustrates the evolution of TPAI values at three perioperative periods: preoperative, 6 months postoperatively (TPAI1R), and 12 months postoperatively (TPAI2R).

Figure 4 illustrates the distribution of TPAI values at three time points: preoperative, 6 months postoperatively (TPAI1R), and 12 months postoperatively (TPAI2R). The NSG had consistently higher TPAI values across all time points compared to the SG. While the NSG showed a slight decline over time, values remained higher than in the SG. In contrast, the SG exhibited persistently low TPAI values across all measurements, with no significant variation over time. These findings indicate that sarcopenic patients maintain consistently lower muscle mass postoperatively, whereas non-sarcopenic patients tend to retain higher TPAI values, with partial recovery by 12 months. These findings should therefore be interpreted as descriptive and hypothesis-generating.

## 4. Discussion

**Figure 5:**
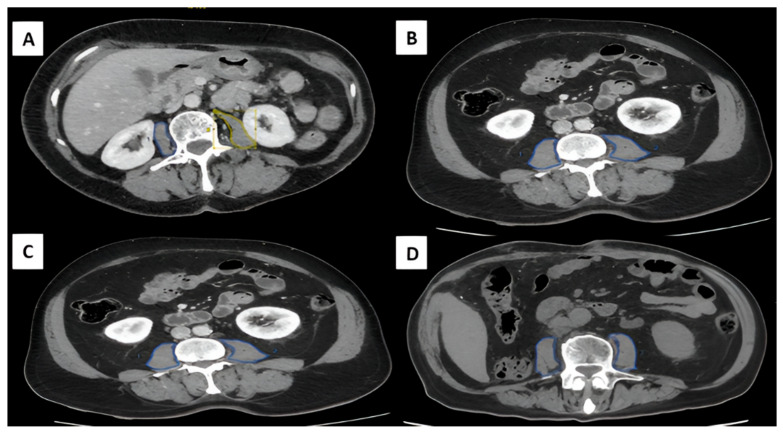
Assessment of sarcopenia using the total psoas area index (TPAI) from axial CT images at the L3 vertebral level. (**A**) Female patient with a normal TPAI of 5.57 cm^2^/m^2^ and height of 1.46 m. (**B**) Female patient with the same height (1.46 m) but a pathological TPAI of 3.85 cm^2^/m^2^ (reference value < 4.98 cm^2^/m^2^). (**C**) Male patient with a normal TPAI of 6.49 cm^2^/m^2^ and height of 1.69 m. (**D**) Male patient with the same height (1.69 m) but a pathological TPAI of 5.45 cm^2^/m^2^ (reference value < 6.04 cm^2^/m^2^). Colored areas (green and blue) indicate the segmented psoas muscle used for TPAI calculation. The images illustrate differences in muscle mass between individuals with and without sarcopenia.

Gastric cancer remains one of the leading causes of cancer-related mortality worldwide, and curative-intent surgical treatment presents numerous challenges, particularly in patients with sarcopenia. In this context, the Total Psoas Area Index (TPAI), measured using computed tomography (CT), has emerged as a valuable tool for assessing muscle mass in patients with gastric cancer and evaluating its impact on surgical outcomes and survival [11] (Figure 5). In contrast to many previous studies that rely solely on CT-derived muscle mass, our study incorporated muscle strength and functional assessment in accordance with EWGSOP2 recommendations, providing a more comprehensive evaluation of sarcopenia. Our findings demonstrate that patients with sarcopenia, defined according to combined EWGSOP2 criteria, have significantly lower overall survival (OS) and disease-free survival (DFS) compared to those without sarcopenia. Although nutritional status was assessed as part of routine clinical practice, it was not included in the present analysis. Given the known interaction between nutrition, muscle mass, and oncological outcomes, future studies should incorporate comprehensive nutritional parameters to better understand their combined impact on prognosis in gastric cancer patients.

As previously described in the literature [6,11], sarcopenia negatively affects surgical outcomes across various diseases, including gastric cancer. TPAI has been recognized as a reliable and reproducible surrogate marker, particularly the psoas muscles at the level of the third lumbar vertebra (L3) [8]. In this study, patients with sarcopenia showed reduced survival and higher rates of postoperative complications, in line with prior research that has identified low muscle mass as a predictor of postoperative complications and poor prognosis in these patients [10,12].

Our analysis revealed a sarcopenia prevalence of 36.7% in our cohort, which is consistent with previous studies reporting rates between 30% and 40% among gastric cancer patients [11,28]. Sarcopenia was associated with a higher rate of postoperative complications such as pneumonia and paralytic ileus, although not all differences reached statistical significance. Nevertheless, the observed trend supports the notion that sarcopenic patients are at higher risk for severe complications, aligning with existing literature that identifies sarcopenia as a predictor of adverse postoperative events [29].

Furthermore, survival analysis showed that sarcopenic patients exhibited a significantly steeper decline in both overall and disease-free survival compared to non-sarcopenic patients. These findings reinforce the prognostic value of TPAI in gastric cancer, as reduced muscle mass may be associated with reduced physiological reserve to withstand surgical stress and postoperative oncologic treatments [9,12].

An important finding of this study is that CT-derived muscle mass (TPAI), as a component of sarcopenia, specifically assessed through the psoas muscle index, showed a strong association with patient survival. The optimal TPAI cut-off values proposed in our cohort are 6.04 for men and 4.98 for women. These thresholds are in line with previous studies, such as that by Sachar et al., which also found that low TPAI is associated with decreased OS and DFS in patients undergoing curative-intent surgery [12]. The TPAI cut-off values were derived using ROC curve analysis within our cohort, which may introduce a risk of overfitting. Therefore, these thresholds should be interpreted with caution and validated in independent populations before broader clinical application. Although sarcopenia showed a significant association with disease-free survival in the univariate analysis, this effect was not maintained after adjustment for confounding variables. This may be explained by the limited sample size and number of events, as well as the strong influence of tumor stage on recurrence outcomes.

Similarly, Zhuang et al. [10] reported higher postoperative complication rates and worse long-term outcomes in sarcopenic patients, further supporting the clinical relevance of TPAI measurement. However, these cut-off values require validation in larger and more diverse cohorts, as imaging techniques and patient-specific factors may influence the results [27].

This study has limitations that should be acknowledged. First, the retrospective design may introduce selection bias and precludes establishing causal relationships between sarcopenia and clinical outcomes. Second, the relatively small sample size (*n* = 79) limits the statistical power of subgroup analyses and may reduce the robustness of some observed associations. Third, the TPAI cut-off values were derived from the study cohort using ROC analysis, which may lead to overfitting and limit generalizability; therefore, external validation in independent populations is required. Fourth, TPAI reflects only the psoas muscle and may not fully represent total skeletal muscle mass. Fifth, although nutritional status was assessed as part of routine clinical care, these variables were not included in the present analysis, which may influence the interpretation of the relationship between muscle mass and outcomes. Finally, the absence of comprehensive multivariable adjustment including all potential confounders and the lack of formal longitudinal statistical analysis of TPAI changes over time should also be considered when interpreting the findings.

Despite these limitations, the use of TPAI thresholds could enhance risk stratification and support more personalized surgical and postoperative management in gastric cancer patients [30]. These findings are consistent with a meta-analysis by Morley et al. [7], which confirmed the association between low TPAI and poor survival, supporting its role as a predictive tool for personalizing curative and adjuvant treatment in this patient population. Nevertheless, TPAI values may vary depending on cancer type and patient characteristics, highlighting the need for standardized thresholds in future research. Our findings should be interpreted in the context of existing evidence. CT-derived muscle mass, as a surrogate of sarcopenia, has been widely reported as a prognostic factor, and our study contributes by providing additional longitudinal data. However, these findings should be interpreted with caution given the limited sample size and the exploratory nature of the analysis.

In addition, we found that sarcopenic patients were significantly more likely to present with advanced tumor stages at diagnosis. Only 20.7% of sarcopenic patients were diagnosed at early stages (I–II), compared to 44% in the non-sarcopenic group. Conversely, 20.7% of sarcopenic patients presented with stage IV disease, compared to only 6% among non-sarcopenic patients.

This distribution suggests a potential link between sarcopenia and late-stage diagnosis, which may be related to delayed healthcare access, lower functional reserve, or more aggressive tumor biology. For instance, Prado et al. reported worse long-term outcomes and higher postoperative morbidity in sarcopenic patients, particularly those with advanced disease stages [31]. This association may partly explain the poorer outcomes observed in this population, as advanced disease stages are typically associated with increased surgical complexity, higher morbidity, and reduced survival.

Regarding surgical intervention, sarcopenic patients were less likely to undergo laparoscopic procedures, possibly reflecting greater case complexity and the relatively small sample size. Minimally invasive approaches are generally preferred in patients with better functional status and less advanced tumors due to lower risks of respiratory and cardiac complications [23].

While our findings did not show significant differences in the overall complication rate, the observed trend toward increased complications in sarcopenic patients suggests that they may benefit from tailored surgical strategies and closer postoperative monitoring.

One of the main implications of this study is that CT-based TPAI measurement may aid in preoperative assessment by identifying patients at higher risk of complications and guiding decisions regarding prehabilitation or functional support strategies following surgery. Such personalized approaches could improve outcomes and optimize resource utilization in the management of gastric cancer patients [21,23].

Finally, although TPAI has shown promise as a prognostic marker, challenges remain regarding the standardization of cut-off values and its applicability across diverse populations. Early identification and management of sarcopenia may be crucial in the comprehensive care of patients with gastric cancer, supporting the need for prospective studies assessing the impact of prehabilitation strategies in this population.

## 5. Conclusions

The Total Psoas Area Index (TPAI), measured using computed tomography, appears to be a useful marker for assessing muscle mass as a component of sarcopenia in patients undergoing curative-intent surgery for gastric cancer.

The presence of sarcopenia, defined according to EWGSOP2 criteria, is associated with reduced overall survival and disease-free survival. Furthermore, sarcopenic patients exhibit a higher risk of postoperative complications, although differences in overall complication rates were not statistically significant in all cases.

Given its significant correlation with clinical outcomes, TPAI has the potential to be a valuable tool for tailoring surgical and postoperative management in patients with gastric cancer. CT-based assessment of muscle mass may help identify high-risk patients and support the implementation of prehabilitation and functional support strategies aimed at improving surgical outcomes and recovery.

Based on our findings, TPAI may be considered a complementary tool in preoperative assessment, although further prospective validation is required before routine clinical implementation. Our proposed TPAI cut-off values for sarcopenia diagnosis are 6.04 for men and 4.98 for women, providing a practical clinical threshold. These findings support the integration of sarcopenia assessment into routine preoperative evaluation to improve risk stratification and guide personalized perioperative management. Although sarcopenia was defined using EWGSOP2 criteria, the relatively small sample size and cohort-specific cut-off values should be considered when interpreting these findings.

Despite its clinical relevance, further prospective studies and external validation are required to establish more definitive cut-off values and to evaluate the effectiveness of sarcopenia management strategies in this context.

## Figures and Tables

**Table 1:** Clinicopathological characteristics.

	N = 79
Age	74 (65, 83)
Sex	
Men	50/79 (63%)
Body Mass Index (BMI)	
Underweight	7 (8.9%)
Normal weight	37 (47%)
Overweight	23 (29%)
Obesity	12 (15%)
Age-adjusted Charlson Comorbidity Index (ACCI)	
Low risk (0–1 points)	1 (1.2%)
Moderate risk (2–3 points)	6 (7.6%)
High risk (4–5 points)	25 (31.6%)
Very High risk (≥6 points)	47 (59.5%)
American Society of Anesthesiologists Physical Status (ASA)	
1	2 (2.5%)
2	37 (47%)
3	32 (41%)
Histological type of cancer	
Adenocarcinoma	79 (100%)
Location of neoplasia	
Antrum	40 (50.6%)
Cardia	14 (17.7%)
Body	18 (22.8%)
Fundus	7 (8.9%)
Tumor stage at diagnosis (cTNM)	
Initial (Stage I and II)	28 (35.4%)
Locally advanced (Stage III)	42 (53.2%)
Advanced (Stage IV)	9 (11.4%)
Neoadjuvant chemotherapy	30 (38%)
Calf diameter	33.4 (5.1)
Dynamometry	25 (8)
SARC-F	
Sarcopenia risk	19 (24%)
No risk of sarcopenia	60 (76%)
Skeletal muscle mass index (SMI)	6.63 (1.84)
No sarcopenia	44 (56%)
Sarcopenia	35 (44%)
Preoperative Total Psoas Area Index (TPAI)	6.33 (4.8, 7.8)
No sarcopenia	50 (63.3%)
Sarcopenia	29 (36.7%)

Results are presented as median (IQR) or frequency (%). Abbreviations: SARC-F. SMI, TPAI.

**Table 2:** Clinicopathological characteristics. Subgroup analysis of total psoas area index (TPAI).

Psoas Total Muscle Area Index (TPAI)	No sarcopenia (*n* = 50)	Sarcopenia (*n* = 29)	*p*-Value
Age	73 (64, 82)	76 (65, 87)	0.2
Sex			0.8
Men	31/50 (62%)	19/29 (65.5%)	
Body Mass Index (BMI)			
Underweight	2 (4%)	5 (17.2%)	<0.001
Normal weight	18 (36%)	19 (65.5%)	
Overweight	18 (36%)	5 (17.2%)	
Obesity	12 (24%)	0 (0%)	
SARC-F			0.02
Sarcopenia risk	8 (16%)	11 (37.9%)	
No risk of sarcopenia	42 (84%)	18 (62.1%)	
Skeletal muscle mass index (SMI)			0.002
No sarcopenia	34 (68%)	10 (34.5%)	
Sarcopenia	16 (32%)	19 (65.5%)	
Age-adjusted Charlson Comorbidity Index (ACCI)			0.001
Low risk (0–1 points)	1 (2%)	0 (0%)	
Moderate risk (2–3 points)	6 (12%)	0 (0%)	
High risk (4–5 points)	15 (30%)	10 (34.5%)	
Very High risk (≥6 points)	28 (56%)	19 (65.5%)	
American Society of Anesthesiologists Physical Status (ASA)			0.6
1	2 (4%)	0 (0%)	
2	24 (48%)	12 (41.4%)	
3	20 (40%)	12 (41.4%)	
4	4 (8%)	4 (8%)	
Histological type of cancer			0.3
Adenocarcinoma	50 (100%)	29 (100%)	
Tumor location			0.1
Antrum	23 (46%)	17 (58.6%)	
Cardia	13 (26%)	1 (6.3%)	
Body	11 (22%)	7 (24.1%)	
Fundus	3 (6%)	4 (8%)	
Tumor stage at diagnosis(cTNM)			0.05
Initial (Stage I and II)	22 (44%)	6 (20.7%)	
Locally advanced (Stage III)	25 (50%)	17 (58.6%)	
Advanced (Stage IV)	3 (6%)	6 (20.7%)	
Neoadjuvant chemotherapy	17 (58.6%)	13 (44.8%)	0.3
Nature of surgical intervention			0.4
Programmed	46 (92%)	21 (72.4%)	
Urgent	4 (8%)	8 (27.6%)	
Type of surgical intervention			0.7
Subtotal gastrectomy	32 (64%)	20 (69%)	
Billroth II	5 (10%)	4 (13.8%)	
Roux-en-Y reconstruction	27 (54%)	16 (55.2%)	
Total gastrectomy	18 (36%)	9 (31%)	
Surgical approach			0.03
Laparoscopic	34 (68%)	13 (44.8%)	
Open	12 (24%)	13 (44.8%)	
Hybrid	4 (8%)	3 (10.4%)	
Type of complications (Clavien–Dindo scale)			0.12
0	22 (44%)	11 (37.9%)	
I	12 (24%)	3 (10.3%)	
II	6 (12%)	6 (20.7%)	
IIIA	3 (6%)	1 (3.4%)	
IIIB	1 (2%)	1 (3.4%)	
IVA	2 (4%)	2 (6.9%)	
IVB	4 (8%)	1 (3.4%)	
V	0 (0%)	4 (13.8%)	
Comprehensive Complications Index (CCI)	17 (9, 25)	30 (24, 36)	0.2
Hospital stay (days)	14 (12, 16)	19 (14, 24)	0.056
Hospital readmission within 3 months postoperatively			0.9

**Table 3:** Cox regression analysis for overall survival.

Variable	HR	95% CI	*p*-Value
Sarcopenia (EWGSOP2-defined)	1.8	1.1–3.2	0.03
Age	1.02	0.98–1.05	0.2
BMI	0.95	0.88–1.02	0.1
Tumor stage (III–IV vs. I–II)	2.5	1.4–4.5	0.002

**Table 4:** Cox Regression Analysis—Disease-Free Survival.

Model	HR	95% CI	*p*-Value
Univariate	2.54	1.24–5.23	0.01
Multivariate *	1.57	0.7–3.51	0.27

* Adjusted for age, sex, and tumor stage.

## Data Availability

The authors confirm that the data supporting the findings of this study are available within the article.

## References

[1] International Agency for Research on Cancer (IARC) World Cancer Report 2014. https://publications.iarc.fr/Non-Series-Publications/World-Cancer-Reports/World-Cancer-Report-2014.

[2] Wu Y., Sun R., Ren S., Zengin G., Li M.Y. (2025). Neuronal reshaping of the tumor microenvironment in tumorigenesis and metastasis: Bench to clinic. Med. Adv..

[3] Song S., Liu Z., Wang Y., Gong B. (2025). Human organoids and their application in tumor models, disease modeling, and tissue engineering. Med. Bull..

[4] Wu J., Zhang P.F., Zeng Y., Hai Y.N., Zhang K.M., Dong S., Xu J.C., Zhang L.L., Wu Z.X., Jiang H. (2025). Effects of FAP+ cancer-associated fibroblasts on anti-PD-1 immunotherapy and CD4+ T cell polarization in gastric cancer. Cancer Drug Resist..

[5] Shi B., Liu S., Chen J., Liu J., Luo Y., Long L., Lan Q., Zhang Y. (2020). Sarcopenia is associated with perioperative outcomes in gastric cancer patients undergoing gastrectomy. Ann. Nutr. Metab..

[6] Cruz-Jentoft A.J., Bahat G., Bauer J., Boirie Y., Bruyère O., Cederholm T., Cooper C., Landi F., Rolland Y., Sayer A.A. (2019). Sarcopenia: Revised European consensus on definition and diagnosis. Age Ageing.

[7] Morley J.E., Abbatecola A.M., Argiles J.M., Baracos V., Bauer J., Bhasin S., Cederholm T., Coats A.J.S., Cummings S.R., Evans W.J. (2011). Sarcopenia with limited mobility: An international consensus. J. Am. Med. Dir. Assoc..

[8] Beaudart C., McCloskey E., Bruyère O., Cesari M., Rolland Y., Rizzoli R., Araujo de Carvalho I., Amuthavalli Thiyagarajan J., Bautmans I., Bertière M.C. (2016). Sarcopenia in daily practice: Assessment and management. BMC Geriatr..

[9] Xu J.Y., Li C., Zhang H., Liu Y., Wei J.M. (2020). Total psoas area index is valuable to assess sarcopenia, sarcopenic overweight/obesity and predict outcomes in patients undergoing open pancreatoduodenectomy. Risk Manag. Healthc. Policy.

[10] Zhuang C.L., Huang D.D., Pang W.Y., Zhou C.J., Wang S.L., Lou N., Ma L.L., Yu Z., Shen X., Yu J. (2016). Sarcopenia is an independent predictor of severe postoperative complications and long-term survival after radical gastrectomy for gastric cancer. Medicine.

[11] Taki Y., Sato S., Nakatani E., Higashizono K., Nagai E., Nishida M., Watanabe M., Ohata K., Kanemoto H., Oba N. (2021). Preoperative skeletal muscle index and visceral-to-subcutaneous fat area ratio are associated with long-term outcomes of elderly gastric cancer patients after gastrectomy. Langenbecks Arch. Surg..

[12] Shachar S.S., Williams G.R., Muss H.B., Nishijima T.F. (2016). Prognostic value of sarcopenia in adults with solid tumours: A meta-analysis and systematic review. Eur. J. Cancer.

[13] De Felice F., Malerba S., Nardone V., Salvestrini V., Calomino N., Testini M., Boccardi V., Desideri I., Gentili C., De Luca R. (2025). Progress and Challenges in Integrating Nutritional Care into Oncology Practice: Results from a National Survey on Behalf of the NutriOnc Research Group. Nutrients.

[14] Charlson M.E., Pompei P., Ales K.L., MacKenzie C.R. (1987). A new method of classifying prognostic comorbidity in longitudinal studies: Development and validation. J. Chronic Dis..

[15] Ajani J.A., In H., Sano T., Gaspar L.E., Erasmus J.J., Tang L.H., Washington M.K., Gerdes H., Kelsen D.P., Mansfield P.F., Amin M.B. (2017). Stomach. AJCC Cancer Staging Manual.

[16] Bahat G., Ozkok S., Kilic C., Karan M.A. (2021). SARC-F questionnaire detects frailty in older adults. J. Nutr. Health Aging.

[17] Chew S.T.H., Tan N.C., Cheong M., Oliver J., Baggs G., Choe Y., How C.H., Chow W.L., Tan C.Y.L., Kwan S.C. (2021). Impact of specialized oral nutritional supplement on clinical, nutritional, and functional outcomes: A randomized, placebo-controlled trial in community-dwelling older adults at risk of malnutrition. Clin. Nutr..

[18] Dindo D., Demartines N., Clavien P.A. (2004). Classification of surgical complications: A new proposal with evaluation in a cohort of 6336 patients and results of a survey. Ann. Surg..

[19] Clavien P.A., Vetter D., Staiger R.D., Slankamenac K., Mehra T., Graf R., Puhan M.A. (2017). The Comprehensive Complication Index (CCI^®^): Added value and clinical perspectives 3 years “down the line”. Ann. Surg..

[20] Carneiro F., Fukayama M., Grabsch H.I., Yasui W. (2019). Gastric adenocarcinoma. WHO Classification of Tumours.

[21] Mourtzakis M., Prado C.M., Lieffers J.R., Reiman T., McCargar L.J., Baracos V.E. (2008). A practical and precise approach to quantification of body composition in cancer patients using computed tomography images acquired during routine care. Appl. Physiol. Nutr. Metab..

[22] Yamashita M., Kamiya K., Matsunaga A., Kitamura T., Hamazaki N., Matsuzawa R., Nozaki K., Tanaka S., Nakamura T., Maekawa E. (2017). Prognostic value of psoas muscle area and density in patients who undergo cardiovascular surgery. Can. J. Cardiol..

[23] Robin X., Turck N., Hainard A., Tiberti N., Lisacek F., Sanchez J.C., Müller M. (2011). pROC: An open-source package for R and S+ to analyze and compare ROC curves. BMC Bioinform..

[24] R Core Team (2024). R: A Language and Environment for Statistical Computing.

[25] Therneau T.M. (2026). A Package for Survival Analysis in R.; R Package Version 3.8-6. https://CRAN.R-project.org/package=survival.

[26] Kassambara A., Kosinski M., Biecek P. (2026). survminer: Drawing Survival Curves Using ggplot2. https://CRAN.R-project.org/package=survminer.

[27] Martin L., Birdsell L., Macdonald N., Reiman T., Clandinin M.T., McCargar L.J., Murphy R., Ghosh S., Sawyer M.B., Baracos V.E. (2013). Cancer cachexia in the age of obesity: Skeletal muscle depletion is a powerful prognostic factor. J. Clin. Oncol..

[28] Argilés J.M., Campos N., Lopez-Pedrosa J.M., Rueda R., Rodriguez-Mañas L. (2016). Skeletal muscle regulates metabolism via interorgan crosstalk: Roles in health and disease. J. Am. Med. Dir. Assoc..

[29] Hamaguchi Y., Kaido T., Okumura S., Fujimoto Y., Ogawa K., Mori A., Hammad A., Tamai Y., Inagaki N., Uemoto S. (2014). Impact of quality as well as quantity of skeletal muscle on outcomes after liver transplantation. Liver Transpl..

[30] Lieffers J.R., Bathe O.F., Fassbender K., Winget M., Baracos V.E. (2012). Sarcopenia is associated with postoperative infection and delayed recovery from colorectal cancer resection. Br. J. Cancer.

[31] Prado C.M., Lieffers J.R., McCargar L.J., Reiman T., Sawyer M.B., Martin L., Baracos V.E. (2008). Prevalence and clinical implications of sarcopenic obesity in patients with solid tumours. Lancet Oncol..

